# Circular bioeconomy: animal by-products from livestock carcass processing

**DOI:** 10.1093/af/vfaf028

**Published:** 2025-09-19

**Authors:** Michael R F Lee, Stewart Ledgard, Lucas Cypriano, Stephen Woodgate, Philippe Becquet

**Affiliations:** School of Sustainable Food and Farming, Harper Adams University, Newport, TF10 8NB, United Kingdom; AgResearch Limited, Ruakura Research Centre, Hamilton 3240, New Zealand; SRTV/S Quadra 701 – Conjunto L- Ed. Assis Chateaubriand, Bloco 1 Sala 114, 70.340-906-Brasilia/DF, Brazil; Beacon Consulting, Kelmarsh Rd., Market Harborough Leicestershire, LE16 9RX, United Kingdom; Philippe Becquet EI, Mulhouse, France

**Implications**

- Livestock carcass production systems (as opposed to dairy and egg production) have as their primary objective to produce meat for human consumption. This production is accompanied by by-products, which are either not consumable by humans or not appreciated (e.g., offals in certain regions).
- By-products from meat production contain valuable components (e.g., protein, fat, minerals), which may be used by or recycled either directly to humans or in livestock feed, following specific processes (called rendering).
- Rendered animal by-products can be used to replace other sources of nutrients such as plant-based proteins (e.g., soybean meal), calcium and phosphorus sources (mined sources), fat (e.g., oil from oilseed), maintaining these nutrients within the food chain and improving sustainability via circularity.
- Advancements in the rendering sector resulting from the Bovine Spongiform Encephalopathy (BSE) crisis have allowed for the safe use of these by-products. However, a higher use of these valuable by-products is required in the context of circular bioeconomy.

## Introduction

The concept of valorization of Animal By-Products (ABP) follows the principle of the ‘Food Waste Hierarchy’ and ‘Value Pyramid’ described by Al Zohairi et al. (2023, Figure 1). Sometimes these concepts are described as a ‘cascading use of biomass’ (Dubois and Gomez San Juan, 2016). The preferred option is source prevention (i.e., avoiding generation of food waste), followed by food recovery where a greater proportion of the animal biomass is used or recovered as human edible food. After food recovery, the value pyramid proposes recycling by-products to produce high-value chemicals and pharmaceutical products, then feed and subsequently food via livestock or social value via pet food, followed by recovery for industrial applications. The latter can include the production of a range of lower value chemicals, and materials such as fertilizers, soap, or biodiesel. The ABP can also be used as a substrate for biodigestion or combusted to produce bioenergy. Finally, when all other options have been exhausted, the remaining ABP can be disposed of through combustion or landfilling.

**Figure 1.**
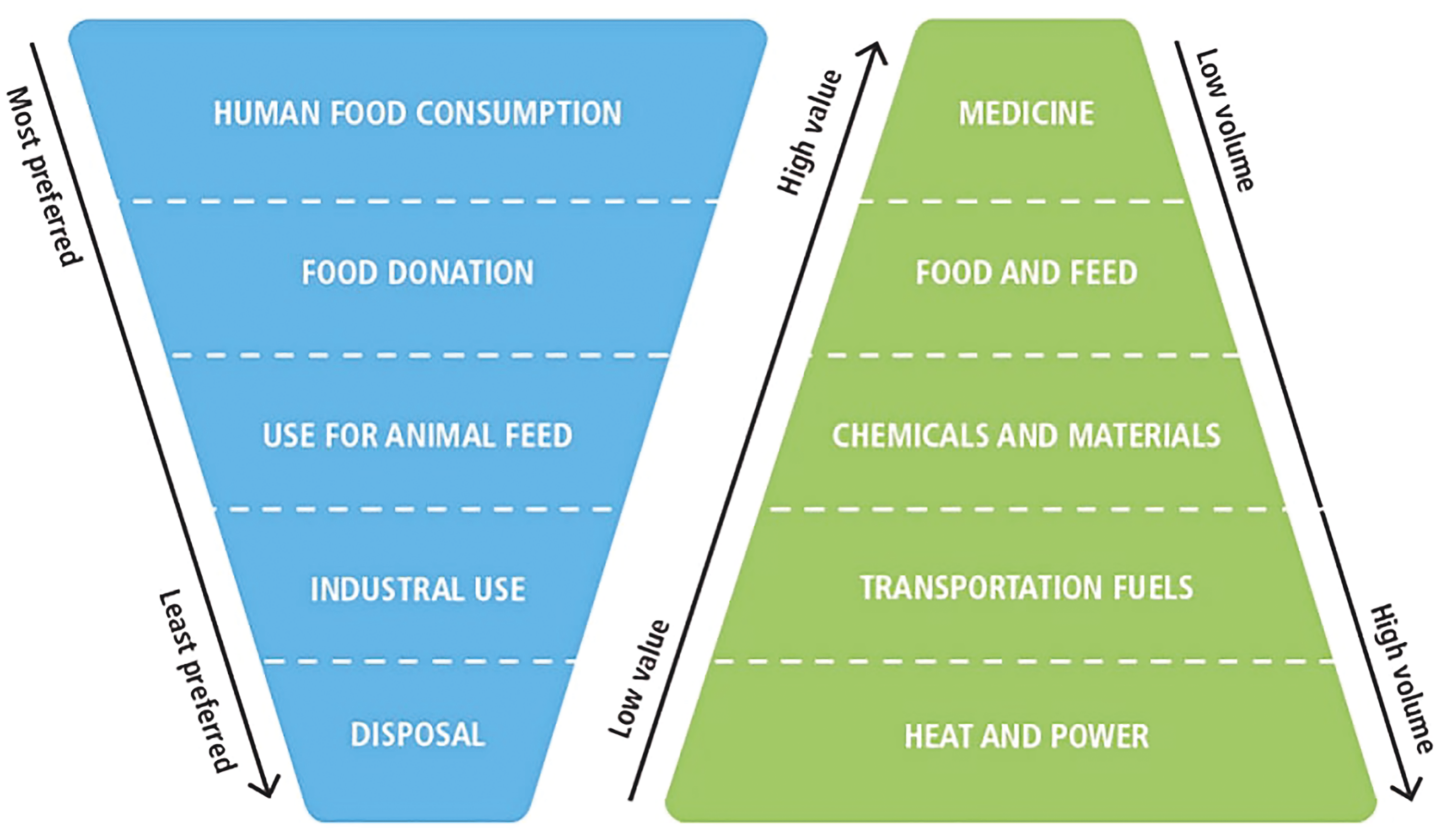
Animal products from abattoirs hierarchy and value pyramid. Source: (Al-Zohairi, Knudsen, and Mogensen, 2023).

Animal-sourced foods are important components of a balanced human diet (Ahmad, Imran, and Hussain, 2018), providing:

- Highly digestible sources of protein (i.e., bioavailable and balanced in essential amino acids).
- High quality (long chain) essential fatty acids (i.e., omega-3 and omega-6).
- Micronutrients, highly bioavailable minerals (e.g., haem iron) and vitamins (including hard-to-source B-vitamins, e.g., B_12_).

Further to the production of animal-sourced food, livestock production systems also provide ecosystem services, manure for fertilization, and multiple by-products with potential to produce a wide range of human edible products, animal feeds, bioenergy, or for higher value uses such as pharmaceuticals, and cosmetics (Wilkinson and Meeker, 2021). In essence, the farming of livestock results in various products that leave the farm for further processing. These include live animals for slaughter and production of meat and offal and their co-products, such as milk, fiber (e.g., wool, mohair, camel hair), and eggs. This paper focuses on the range of by-products from livestock carcasses via rendering and subsequent potential in a circular bioeconomy.

### By-products of livestock carcass processing

**Figure 2.**
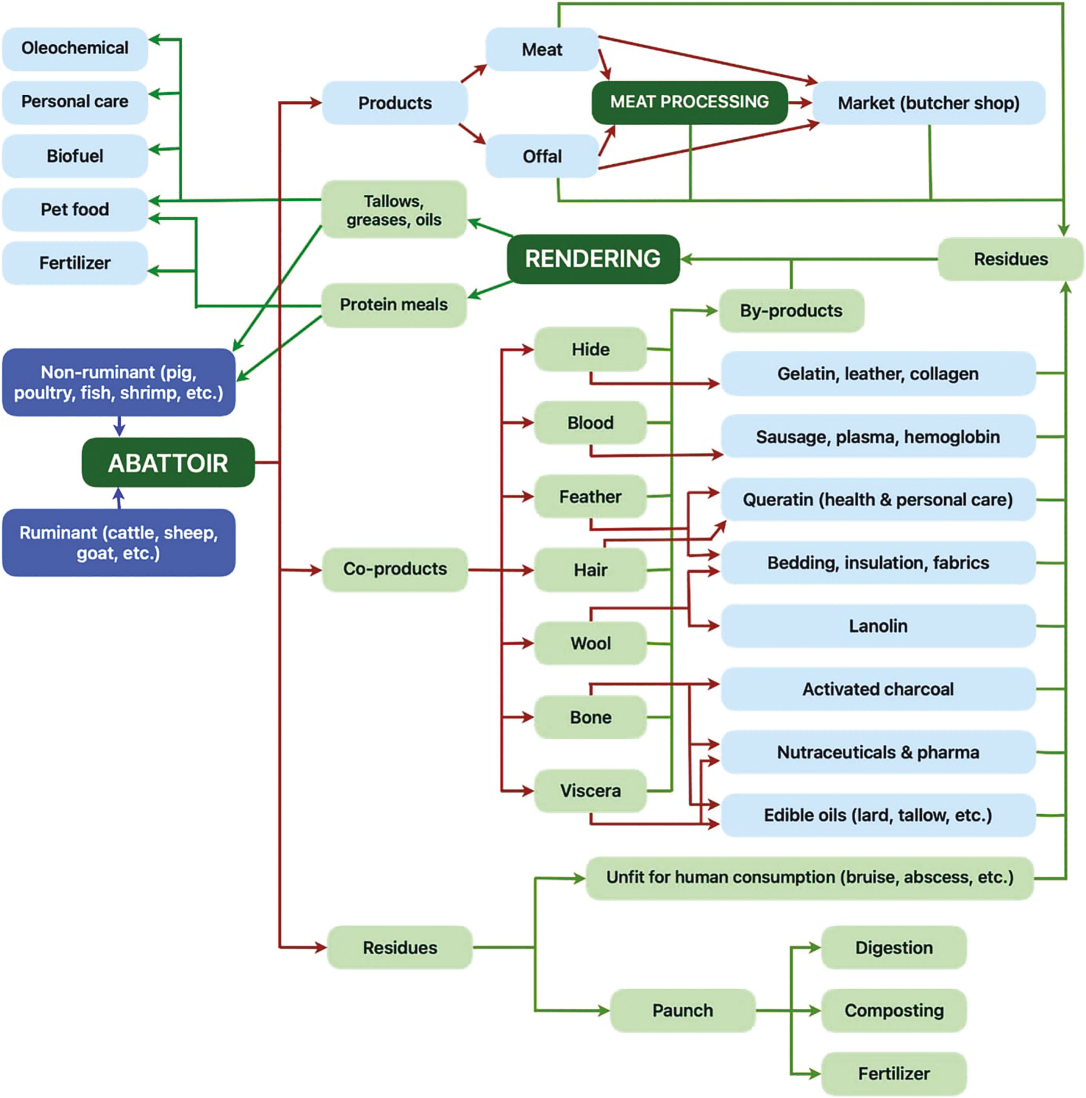
Description of multiple co-products, residues generated from abattoirs and rendering plants, illustrating various options for upcycling and increased circularity. Source: Scientific Advisory Panel, World Renderers Organization, 2023. In dark blue, source of the products and co-products, in light blue, high value products, in light green, by-products, and in dark green and upper case, processes.

Livestock is processed for edible products (meat and offal), but there are also multiple other by-products, residuals, and wastes with the potential to generate alternative products (Figure 2). Redirection of these products towards alternative uses and away from waste streams is a key component of achieving circularity, while in concert considering their safety. Rendering is an important step in livestock processing, as it enables animals that have not been approved for livestock processing for human consumption (e.g., livestock failing inspection of carcass or carcass quality imperfection) or human inedible products (e.g., bones, feathers) from abattoirs to be upcycled into a range of safe and valuable by-products (Wilkinson and Meeker, 2021; Figure 2). For example, paunch (i.e., gastrointestinal content) from abattoirs is predominantly used as a source of energy in abattoirs (Karlis, Prescicce, Giner Santonja, Brinkmann, and Roudier, 2024) or to generate compost as a fertilizer.

An array of high-value ABP (supporting the principle of the value pyramid, Figure 1) are produced for applications in biobased industries, such as food, pharmaceutical, and cosmetic industries (e.g., heparin, glucosamine, gelatin, chondroitin; Table 1; Toldrà et al., 2021). Pharmaceutical grade blood collection is common in abattoirs for plasma or serum. One company in New Zealand uses over two (2) million liters of blood, which is extracted for blood products including serum for cell culture and biomedical purposes, as well as bovine serum albumin (e.g., used as transport carrier for various drugs) and pro-thrombin (i.e., for controlling blood coagulation). In 2022, exports of blood products and glands from processed cattle and sheep returned over US$130 million or the equivalent of over 2% of the total returns in New Zealand beef and sheep meat (MIA, 2022). This collection and processing of blood into high-value pharmaceutical products is an example of upcycling to the top of the value pyramid (Figure 1). Similarly, heparin is a widely used pharmaceutical anticoagulant for preventing blood clotting in medical procedures, which is derived from the mucosal tissue of livestock, particularly pig intestines (Middeldorp, 2008). Innovative and new green extractive techniques have been developed for further extraction of important molecules in co-products of the rendering industry such as pulse electric, microwave, extrusion, or ultrasound assisted extraction, high hydrostatic pressure extraction, supercritical fluid extraction, pressurize liquid extraction, subcritical liquid extraction, membrane separation technologies, fermentation and enzymatic extraction (Bruno, Anta Akouan Ekorong, Karlal, Catherine, and Kudre, 2019).

**Table 1.** A summary of the main high-value animal-based products

Product	Source	Main uses	Some key references
Keratin	Annually, global keratinous products—9 to 13.2 million tons of which: -1–2 million tons of sheep wool -8–10 million tons poultry feathers -1.2 million tons of horns and hooves	Biomaterials with a wide range of uses: -pharmaceuticals industry -tissue engineering -automotive -aerospace industry	(Chukwunonso Ossai, Shahul Hamid, and Hassan, 2022)
Gelatin	Pure collagenous protein. Hides from swine and cattle, fish skins, and bones of swine and cattle are primary sources of gelatin. In the EU, some sources of gelatin are prohibited for use as a feed ingredient, while in other countries, fish is a major source of gelatin.	Two types of gelatin are produced: - edible—regulated by food standards, also used in feed. - pharmaceutical—regulated by the official pharmacopeia.	(Gündem and Tarhan, 2020)
Protein hydrolysates	ABP proteins are hydrolyzed by proteases, high hydrostatic pressure, ultrasound, or other methods to obtain free amino acids and bioactive peptides.	-Functional feeds are particularly important for growing animals and those suffering from inflammation and infections. -Media for cell culture. -Treatment for the harmful side-effects of non-steroidal anti-inflammatory drugs in farm animals or pets. -Treatment of osteoarthritis in pets.	(Martinez-Alvarez, Chamorro, and Brenes, 2015)
Chondroitin	A complex sugar produced from processed cartilage, usually derived from cattle trachea and pig ears, but also from animal joints.	Treatment of arthritis in both humans and pets	(Bhathal, Spryszak, Louizos, and Frankel, 2017)

As described in Figure 2, the slaughtering of livestock leads to a large variety of products, some of them consumed as food by humans, while others (co-products) may provide valuable materials, such as nutraceuticals, clothing, and building materials (e.g., insulation) and be used by biobased industries. Some residues of meat production and of the biobased industries that are unsuitable for human consumption can be upscaled by rendering (in green boxes). In Europe and North America (United States of America and Canada), about 49.5 million tons of by-products and residues are produced by abattoirs, mostly from non-edible parts of the carcass rather than other causes (e.g., fallen stock or condemned carcasses).

Variations exist between various countries on the type of co-products, as global consumers may have different consumption models, e.g., in Europe, offal and blood are not largely consumed, in contrast to Asia or Africa. Thus, the use of co-products may be different between countries. This necessitates having a local evaluation of the resources and their potential use.

Protein (and bones) meal and rendered fats are the two (2) key ingredients produced through rendering processes.

In line with the hierarchy and value pyramid (Figure 1), the preferred use of rendered product should be in livestock feed to keep the nutrients included in these products within the food chain, while ensuring the safety of the food chain. Further upcycling may be produced with the remaining volume.

### Use of rendered by-products in livestock feed and pet food

Dietary protein is a vital component of any animal feed as it supports bodily functions, growth, muscle development, and other production processes (e.g., milk and eggs). It is also the costliest component of the ration, financially and often environmentally (Azarkamand et al., 2024). Currently, soybean meal is the most common non-forage protein source fed to livestock, depending on market conditions and availability. More sustainable circular protein sources are critically needed. Protein meals from rendered ABP represent a potentially valuable source of protein with digestibility between 75% and 94% and a high biological value (up to 8.7% lysine and 1.4% methionine), depending on the sourced meal (INRAE CIRAD AFZ and FAO, 2025). Research indicates that many of these sources could be substituted for soybean meal in the diet of pigs, poultry, and aquaculture. Protein meal can account for 5% to 25% of the diets of poultry and pigs, supplying one third of the dietary protein (Leiva et al., 2018). Studies with pigs, where soybean meal was replaced by protein meal, have had variable responses. (Shelton et al., 2001) reported lower performance of growing finishing pigs offered protein meal as compared with soybean meal. In contrast, (Gottlob et al., 2004) reported improved pigs’ average daily gain when protein meal constituted up to 5% of the diet, with it performing as well as soybean meal at higher incorporation rates. In poultry, the replacement of soybean meal and dicalcium phosphate with protein meal at 2%, 4%, or 6% had no effect on egg mass (i.e., more eggs with lower weight) and no change in feed conversion rate, body weight, or mortality (Bozkurt, Alçiçek, and Cabuk, 2004). In aquaculture, the suitability of protein meal to replace soybean meal has also been exemplified in a range of fish species (*Trachinotos carolinus* L. [Rossi and Davis, 2014]; *Spraus aurata* [Moutinho et al., 2017]; *Ictalurus punctatu* [Mohsen and Lovell, 1990]). However, due to the nature of protein meal production, its composition, quality, and nutritional value as a sustainable feed ingredient are related to the nature of the material from which it is derived (Hendriks et al., 2002). In addition, the level of protein meal incorporated in livestock diets may also be limited by its mineral content. Solà-Oriol, Roura, and Torrallardona (2011) reported that feeds with 5% protein meal were preferred over soybean meal by pigs. However, values greater than 5% reduced feed intake, probably due to the high mineral content. Dietary minerals (especially calcium and phosphorus) are important for skeletal development, milk composition, and eggshell production. Currently, mined phosphate rock and limestone are used as sources of phosphorus and calcium, but protein meals and bone meals are also sources of highly digestible calcium and phosphorus. Bozkurt et al. (2004) showed that egg quality (specific gravity and Haugh unit) and eggshell integrity (less cracked–broken eggs) were improved when protein meal replaced dicalcium phosphate in the diet of layers.

As a provider of high-quality protein, vitamins, and minerals, protein meal is a vital ingredient in pet foods, which cannot be easily replaced by plant-based proteins. This is especially true for cats as carnivores that have a specific requirement for taurine, which is lacking in all plant-based protein. In addition to their nutritional value, protein meals have been shown to improve the palatability of pet foods (Boskot, 2009).

Rendered fats are also a vital ingredient in feed, as well as for the food sector, as depending on the production system, they may offset environmental impacts caused by the growth of the palm and soybean oil industries. Palm oil replaced rendered fats in many food items in response to human health concerns (C16:0 and C18:0). However, it has been shown that C16:0, which is in higher concentration in palm oil than rendered fats, raises LDL cholesterol, whereas C18:0 (i.e., predominantly saturated fatty acids in rendered fats), does not raise LDL cholesterol, possibly due to its rapid conversion to oleic acid in the body (Grundy, 2013).

With growing interest and financial returns in the pet market, ABP are also being included as a functional ingredient in pet foods either as a nutraceutical (Table 1) or to improve digestibility (e.g., pancreatin, extracted from pigs’ pancreases (Bampidis et al. 2023). In addition, other ABP are used as pet treats, such as pigs’ ears, raw hide, and bones (Martinez-Alvarez, Chamorro, and Brenes, 2015).

### Limitation of use

Bovine Spongiform Encephalopathy (BSE), a fatal neurodegenerative disease in cattle caused by prions, was first identified in the United Kingdom in the 1980s and rapidly spread due to the use of infected ruminant-derived meat and bone meal in cattle feed. Woodgate and Wilkinson (2021) described how changes in the rendering sector in the 1980s, driven by economics, resulted in a failure to deactivate the causative prion proteins associated with transmissible spongiform encephalopathies (e.g., BSE, scrapie, Creutzfeld-Jacob disease). Subsequent transmissible spongiform encephalopathy deactivation trials showed that a traditional rendering system, including a hyperbaric pressure stage (three bars for 20 minutes), resulted in the inactivation of transmissible spongiform encephalopathy to below detectable levels (Woodgate and Wilkinson, 2021). The emergence of BSE led European countries to restrict the use of specified risk materials, including all protein meal products from all livestock species for use in feed (Woodgate and Wilkinson, 2021), to further prevent the BSE transmission. Moreover, the World Organization of Animal Health (WOAH) decided to develop strict precautionary measures to control the disease that focused on avoiding cattle being fed with ruminant protein meals (WOAH, 2024a). Over time, the implementation of these measures significantly reduced the number of classical BSE cases worldwide.

WOAH established guidelines to mitigate risks of BSE (WOAH, 2024b). Regarding the rendered products, the major recommendations are focused on:

-Feed ban: bovines should not be fed ruminant-derived protein meals to prevent transmission.

-Safe commodities: bovine tallow, with a maximum of 0.15% insoluble impurities, are considered safe.

-Rendering and feed manufacturing: good manufacturing practices in rendering plants and feed mills are critical to avoiding cross-contamination of bovine feed with ruminant-derived protein meals. Preventive measures must be in place to ensure that ruminant-derived protein is not introduced into bovine feed.

-Surveillance and risk categorization: countries are classified on their BSE status:

◦ Negligible risk: no cases or effective control measures in place.
◦ Controlled risk: past cases or effective mitigation strategies in place for less than eight (8) years.
◦ Undetermined risk: Insufficient information or inadequate control measures.

The European Union (EU) implemented a strict strategy to control the spread of BSE, establishing a system for categorizing ABP into three (3) risk categories (Table 2). The typical composition of meat and co-products and their subsequent categories in four (4) main terrestrial species is provided in Table 3. Additionally, the EU imposed further strict restrictions on the use of processed animal proteins in livestock feed, prohibiting, for example, the feeding of non-ruminant (i.e., swine and poultry) with ruminant-derived proteins. Under the current EU legislative regime, only specific protein meals such as poultry (650 kilotons per annum), feather (220 kilotons per annum), or pig (420 kilotons per annum) meal can be used as feed ingredients. Subsequently, about 1,300 kilotons per annum of ruminant or multispecies meals are restricted to use only as pet foods, offering a potential supply of proteins from the rendering industry, as the cause of BSE is now understood and prion deactivation confirmed (Woodgate and Wilkinson, 2021).

**Table 2.** Classification of the categories of animal co-products, based on Regulation (EC) No 1069/2009

Category	Definition
1—Highest risk	-Carcasses and all body parts of animals suspected of being infected with TSE (transmissible spongiform encephalopathy) -Carcasses of wild animals suspected of being infected with a disease that humans or animals could contract. -Carcasses of animals used in experiments. -Parts of animals that are contaminated due to illegal treatments. -International catering waste -Carcasses and body parts from zoo and circus animals or pets -Specified risk material (brains and spinal cords of ruminants)
2—High risk	-Animals rejected from abattoirs due to having infectious diseases. -Carcasses containing residues above the maximum limit from authorized treatments (e.g., medicines, research) -Unhatched poultry that has died in its shell. -Carcasses of animals killed for disease control purposes. -Carcasses of dead livestock -Manure (lairage) -Paunch (digestive tract contents)
3—Low risk	-Carcasses or body parts deemed fit for humans to eat, at an abattoir. -Products or foods of animal origin originally meant for human consumption but withdrawn for commercial reasons, not because they are unfit to eat -There are no restrictions for the use of poultry and swine processed proteins in feed for aquaculture.

Source: www.gov.uk.

**Table 3.** Typical composition of meat and animal co-products from livestock farmed for food and their categorization in Europe (Woodgate, 2023)

	Cattle	Sheep	Pigs	Poultry
Description	Meat and meat products intended for human consumption			
% animal liveweight	60	55	70	68
Description	Animal co-products NOT intended for human consumption			
% animal liveweight	40	45	30	32
% ABP Category 1***	3	5	0	0
% ABP Category 2***	17	12	11	3
% ABP Category 3***	20	28	19	29

^***^See Table 2 for definitions of category 1–3.

Outside of the EU, most countries have not adopted this specific classification system and restrictions. Instead, many follow the WOAH recommendations, which provide guidelines for the prevention and control of every transboundary disease, including BSE. A good example of the implementation of the WOAH Code is Canada, which, after recorded cases of classical BSE, prohibited the use of specific high-risk materials in feed and introduced rigorous surveillance measures. These actions effectively eliminated the occurrence of classical BSE in the country, without the need to adopt the EU classification system. Canada is now recognized as a country with negligible risk for BSE. The list of countries’ status can be found at WOAH website (https://www.woah.org/en/disease/bovine-spongiform-encephalopathy/).

Furthermore, in most parts of the world, the use of ruminant-derived or non-ruminant-derived rendered meals and fats has never been prohibited in the diets of monogastric livestock species (poultry, pigs, fish, crustaceans). In some countries, the sole restriction on ruminant feeding is ruminant-derived protein meals, while the other meals, such as fish meal, feather meal, and blood meal, can be used in their diets since those rendered meals pose no risk of amplification of classical BSE (Meeker, 2006).

### Other uses of rendered products

Rendered meals, including bone meal, blood meal, and feather meal, have been used in agriculture for centuries as natural fertilizers and soil amendments. These by-products of the meat processing industry offer sustainable alternatives to synthetic fertilizers, enhancing soil fertility and promoting plant growth.

The application of ABP in agriculture dates to ancient civilizations. Even before the rendering industry emerged, farmers recognized the fertilizing properties of animal products, incorporating them into soil to enhance crop yields (Tietz and von Minckwitz, 2023). Fish-based fertilizers were used during the Edo period in Japan, where by-products from oil extraction of fish (e.g., sardines and herring) served as effective fertilizers, boosting the market economy (Animoto, 2024). During the Industrial Revolution, the systematic production of bone meals began, with abattoirs producing large quantities of bones and residuals. These residuals processed into bone meal or meat and bone meal were primarily used as fertilizers. This practice underscored the value placed on natural fertilizers in enhancing soil fertility (Meeker, 2006). The use of rendered meals as fertilizers was predominant until 1901, when Professor C.S. Plumb from Purdue University conducted an experiment where he added animal protein meal to the corn-based diet fed to pigs. This supplementation significantly accelerated growth and allowed the pigs to be ready for market in less than seven (7) months, elucidating a further alternative for animal protein meal other than fertilizer (Meeker, 2006).

Today, animal-based meals, not used as feeds or higher-value products, continue to play a significant role in sustainable agriculture due to their nutrient content and slow-release properties, contributing to long-term soil health.

- **Bone meal:** rich in phosphorus and calcium, bone meal serves as a slow-release fertilizer providing essential nutrients that support root development and flowering in plants. Its solubility in water requires microbial activity or soil acidity to break it down, releasing nutrients over several months ([Meeker, 2006]; [Jayathilakan, Sultana, Radhakrishna, and Bawa, 2012]).
- **Blood meal:** As a high-nitrogen organic fertilizer, blood meal promotes vigorous vegetative growth. Derived from the dried animal blood, it offers one of the highest organic sources of nitrogen, making it particularly beneficial for leafy crops (Jayathilakan, Sultana, Radhakrishna, and Bawa, 2012).
- **Feather meal:** Processed from poultry feathers, feather meal is a valuable nitrogen source. Its slow decomposition rate provides a steady nitrogen supply, enhancing soil fertility over time (Hartz and Johnstone, 2006).

If not used as a feed ingredient, the incorporation of rendered meals into the soil offers several benefits that align with sustainable agricultural goals:

- **Nutrient recycling:** Utilizing ABP, extracted from pigs' pancre the soil, reducing the need for synthetic fertilizers ( Koutsoumanis et al., 2021).
- **Soil health improvement:** These organic fertilizers enhance soil structure, increase organic matter content, and stimulate microbial activity, regenerating soil quality. Such improvements lead to better nutrient availability and water retention, fostering a healthier soil ecosystem (Giacometti et al., 2021).
- **Environmental benefits:** Replacing chemical fertilizers with ABP can lower greenhouse gas emissions and reduce environmental pollution associated with synthetic fertilizer production and application (Jayathilakan, Sultana, Radhakrishna, and Bawa, 2012).

Using a circular footprint formula, which accounts for the impact between system, Kyttä et al. (2021) calculated that greenhouse gas emissions were reduced by about 50% per ton of oat grain, when using a nitrogen-rich meal fertilizer as compared with inorganic nitrogen fertilizer. Ash-based fertilizers are also high in minerals, especially phytoavailable phosphorus, making it a sustainable alternative to rock phosphate (Piash, Uemura, Itoh, and Iwabuchi, 2023).

Rendered fats have a whole array of uses other than food ingredients (lard), feed and pet-food ingredients, such as biodiesel, renewable diesel, and sustainable aviation fuel. In Brazil, the National Agency for Petrol, Gas and Biofuels informs that a total of 773,000 m^3^ of rendered fats were used in 2024 as a raw material for their national biodiesel industry (Painel Dinâmico de Produtores de Biodiesel, 2025).

Another important use of rendered products was the manufacture of soap in the oleochemical industry. However, the proportion of rendered fats used in the manufacture of soap by saponification has declined over the last twenty-five (25) years, driven by a preference for liquid soaps produced from plant-based oils, such as rapeseed and palm (Cerone and Smith, 2021). This transition to palm oil soaps is now being questioned, given a wider understanding of the environmental impact of palm oil plantation on biodiversity loss. Consequently, the use of rendered fats may be a more sustainable choice due to its biocircular credentials. Rendered fat soap also naturally contains vitamin A, D, E, K, and B_12_, which reportedly contribute to skin health and appearance.

### Environmental benefits and risks

Rendering is a critical step for utilizing inedible or unsuitable products for the human consumer and contributing to regenerative agriculture. It is a key component of the circularity of livestock production systems, keeping the highly valuable nutrients they contain in further food production, and can avoid issues with sending unwanted materials to landfill, where they increase the risk of methane emission and groundwater contamination. It also allows for upcycling products that may otherwise be wasted or restricted to uses, lower in the hierarchy pyramid, such as bioenergy and fertilizer of compost (Koukouna and Blonk, 2020). Based on an Attributional Life Cycle Assessment, (Koukouna and Blonk, 2020) demonstrated that the carbon footprint of rendered products varied between products, but were in the same range or lower than the carbon footprint of other protein plant sources (e.g., soybean meal), when evaluated on the basis of the protein content of the feed ingredient. Land Use Change induced by the production of soybeans largely impacted the carbon footprint of the latter feed ingredient.

Hence, the use of rendered animal products may reduce the carbon footprint of feed production by keeping nutrients in the food chain. They achieve a similar nutrient profile and thus similar production levels as plant-based production level. Following the hierarchy in Figure 1, co-products not used for animal feeds may be used as fertilizers, reducing the reliance on inorganic, fossil-based fertilizers, further reducing the food production system’s carbon footprint.

Abattoirs, however, also produce wastewater during plant cleaning, animal excreta in yards prior to slaughter, and liquids (e.g., blood, paunch). If these are voided in waterways, they can contribute to eutrophication. In many parts of the world (Europe, Australia, New Zealand, and the Americas), there are strict regulations limiting wastewater entering waterways to minimize eutrophication. Wastewater from processing plants (e.g., from cleaning and washing down yards used for holding animals prior to slaughter) is commonly applied to agricultural land as a substitute for chemical fertilizers. However, even greater benefits could be derived if it were stored in ponds prior to land application and the methane emitted was captured for bioenergy. Alternatively, other nutrient recovery and recycling options could be used for valuable elements, such as nitrogen and phosphorus. Once captured, they could be used as fertilizers in agricultural systems, which reduces the need for synthetic fertilizers, conserves resources and reduces nutrient pollution of water bodies.

## Conclusion

The processing of animals for meat also produces a myriad of other important co-products and by-products. Some of these can be upcycled for human consumption, while others have high value, such as for pharmaceuticals and biomedical purposes, as well as for essential human needs (clothing, housing). Rendering is critical for utilizing and upcycling the residues and non-utilized animal tissues.

Rendering ABP from meat production allows the maintenance of nutrients in the food chain, using the concept of circular bioeconomy. It keeps high-quality protein, minerals, and fats prioritizing their use in feed for livestock production systems, followed by pet foods, lower-value chemicals, fertilizers, and finally for bioenergy. However, current limitations, mainly linked to regulatory restrictions in some countries, following the BSE epidemic, reduce the potential of these rendered products for the circular bioeconomy. Applying WOAH’s available recommendations (proper slaughtering and rendering processes, good feeding practices, and surveillance) eliminates the risk of prions. At the same time, maintaining the nutritional value of rendered products and sourcing them from animals declared safe for human consumption would enhance the potential for increased use of these valuable nutrients in livestock production.

This is a clear application of the circular bioeconomy principle.

## About the Authors

**Professor Michael Lee** FRSA FRSB FRASE ARAgS is an expert in sustainable livestock systems, defining their role in securing global food security at the same time as protecting environmental health (Livestock’s role in human and planetary health). He graduated with first-class honors in Animal Science from University of Wales, Aberystwyth in 1997 and gained a PhD in ruminant nutrition (protein and energy metabolism) from the University of Aberdeen in 2001, followed by a Postgraduate Certificate for teaching in higher education from Aberystwyth University in 2012. In November 2020, Michael moved from Rothamsted Research and the University of Bristol (where he held a joint position) to his current position as Deputy Vice Chancellor of Harper Adams University, England’s Premier specialist agriculture and land use University. He has published over 375 research articles and papers, including articles in Nature and Science. He served as President of the British Society of Animal Science from 2018 to 2022 and was a Trustee of the society for 12 years. In August 2019, he was elected President of the European Federation of Animal Science Livestock Farming Systems Commission. He represents the UK on Animal Task Force, a Public-Private think-tank informing the EU parliament in Brussels, and is co-chair of the UK Universities Climate Network—Net Zero group. In 2023, he was selected to join a Technical Advisory Group of the UN’s Food and Agriculture Organization (FAO). Prof Lee also sits on the BBC’s Rural Affairs Committee, the Agriculture Advisory Group convened by the UK’s Climate Change Committee (CCC), and the Scientific Council of the World Farmers Organization (WFO).

**Professor Stewart Ledgard** is a Principal Scientist with AgResearch (a Crown Research Institute) and an adjunct Professor with the Life Cycle Management Center at Massey University in New Zealand. His PhD covered nitrogen cycling in agricultural systems and this research area expanded to cover impacts on water quality, resource use efficiency, and environmental emissions through agricultural supply chains. Stewart’s expertise covers detailed research in ruminant livestock farm systems and supply chains, with a focus on reducing emissions and increasing circularity, utilizing life cycle assessment. He has published over 280 research papers and 10 book chapters, as well as working closely with New Zealand farming, policy, and industry groups on reducing agricultural impacts on the environment and from livestock supply chains to international markets. Stewart has strong international collaborations and has regularly contributed to various guidelines and tools developed by the International Dairy Federation and the Livestock Environmental Assessment and Performance (LEAP) partnership.

**Stephen Woodgate** is a recently retired consultant in the animal by-product industry. He was previously technical director of the largest UK rendering company, and technical director of the UK, European, and World rendering associations. Stephen also served as president of the World Renderers Organization (WRO), where he was actively involved with international organizations such as the FAO and the World Organization for Animal Health (WoAH). In the last 25 years, Stephen has been actively involved with the strategic development of the animal by-products sector, including the use of research to steer legislation. During the same period, he drew on his experience over 40 years in the industry to complete his PhD by publications at Harper Adams University.

**Lucas Cypriano,** Zootechnician (Animal Science), worked from 1998 to 2003 as a nutritionist for poultry and swine, formulating feed and premixes, as well as implementing and managing the 5S and ISO 9001 systems. From 2003 to 2011, he worked with feed additives, such as phytogenics and preservatives for rendered meals and fats. Since 2011, Lucas has led the Technical Department of ABRA (Brazilian Renderers Association), overseeing regulatory matters, hygienic manufacturing programs, HACCP plans, and rendering project consultancy. With the WRO (World Renderers Organization), he has been the Brazilian Delegate since 2012 and a member of the Scientific Advisory Panel since 2014. Having held the positions of vice president and president within the WRO, since 2023 is the Technical Director of the WRO and serves as its delegate to IFIF (International Feed Industry Federation), WOAH (World Organization for Animal Health, formerly OIE), FAO (Food and Agriculture Organization of the United Nations), and LEAP (Livestock Environmental Assessment and Performance Partnership of FAO), where he is a member of the Steering Committee.

**Philippe Becquet** is a consultant for regulatory and sustainability topics. He provides advice to the feed industry. Agronomist and animal nutritionist by background, Philippe Becquet has worked for more than forty years in the feed industry at various positions and in various companies, accumulating experience and expertise in livestock production systems and the feed industry. He has also been active in feed industry associations such as IFIF (International Feed Industry Federation) and has contributed as an expert in the development of the Livestock Environmental Assessment and Performance (LEAP) guidelines on feed additives, the report on methane, and more recently to the report on the role of livestock in circular bioeconomy.
